# Leisure activity participation, health status, and subjective well-being of adult women: a cross-sectional study from a life cycle perspective in China

**DOI:** 10.3389/fpubh.2025.1570705

**Published:** 2025-06-18

**Authors:** Nan Xiao, Yuhua Ma, Yang Zhao, Pengfei Li, Zhaoxiang Li, Jie Yang

**Affiliations:** ^1^Shandong Sport University, Jinan, China; ^2^Qufu Normal University, Qufu, China; ^3^City University of Hong Kong, Kowloon, Hong Kong SAR, China

**Keywords:** life cycle, leisure activities, subjective well-being, health status, women’s development

## Abstract

**Objective:**

Based on life cycle theory, this study aims to explore the changes in leisure activity participation, health status, and subjective well-being among women at different life stages, and to investigate the underlying mechanisms. Additionally, the study examines the moderating effects of demographic characteristics.

**Methods:**

This study used data from the China General Social Survey (CGSS) and employed a cross-sectional research design to compare data from women in early adulthood, early maturity, and late maturity. Stepwise regression and mediation analysis were applied to systematically examine the complex relationships between leisure activity participation, health status, and subjective well-being.

**Results:**

The results indicate that women in early adulthood report significantly higher levels of subjective well-being and health status than those in early and late maturity stages. Furthermore, leisure activity participation showed significant changes across the life cycle. Stepwise regression analysis revealed that the impact of leisure activity participation on subjective well-being varied across different life stages, but health status remained a key determinant of well-being. Mediation analysis further confirmed that health status partially mediated the relationship between leisure activity participation and subjective well-being.

**Conclusion:**

This study provides an in-depth examination of the dynamic changes in women’s subjective well-being across the life cycle and highlights the strategic importance of leisure activities in enhancing women’s quality of life. Future research could employ longitudinal tracking designs to explore the causal mechanisms between these variables in greater depth, while also considering the multifaceted influences of cultural background and psychosocial factors.

## 1 Introduction

The relationship between leisure activity participation (LAP), health status, and subjective well-being (SWB) has gained increasing scholarly attention, particularly regarding gender differences and life-cycle stages (1–3). Leisure activities enhance physical health, alleviate stress, and increase life satisfaction (1, 4), making them crucial components of overall quality of life. For women, participation in leisure activities is influenced not only by health status but also by changing social roles across different life stages—early adulthood, middle age, and late adulthood (2).

Research has consistently demonstrated that leisure activities positively impact both physical and mental health. Regular participation in light to moderate-intensity leisure activities reduces the risk of chronic diseases while improving cardiovascular function and immune system performance (5). Leisure engagement also alleviates stress and reduces symptoms of anxiety and depression, with studies showing that consistent participation markedly improves emotional regulation (6). This aspect is particularly important for women, who often face substantial pressures from family, work, and social expectations.

Gender differences in leisure participation are significant, especially within Chinese cultural contexts where traditional gender roles influence women’s activity choices (7). Women typically prefer cultural or social activities and often have more limited leisure time due to childcare and household responsibilities (8). As women age, their leisure choices evolve in response to changing role pressures and health concerns (3).

Leisure activities impact subjective well-being through multiple pathways. They enhance life satisfaction by providing relaxation and enjoyment away from daily demands (9), foster self-actualization through personal achievement (10), and offer crucial psychological respite for women balancing multiple responsibilities (11). Health status serves as a critical mediator in this relationship, as physical health directly influences emotional states and life satisfaction. Individuals in better health typically report higher quality of life, with health conditions significantly affecting women’s emotional state and well-being during transitions in family and social roles.

The life course perspective provides a valuable framework for understanding these relationships across different age groups. Women in early adulthood face career development and family formation pressures, middle-aged women navigate increased family and workplace responsibilities, and women in late adulthood often modify their leisure pursuits due to health changes and shifting social networks (3). Despite extensive research on these topics, most studies have focused on Western contexts, with insufficient attention to Chinese women’s experiences (7). Additionally, many studies fail to systematically examine how life stages influence the relationships between leisure activities, health, and well-being (12).

In the context of China’s rapid social transformation, modern Chinese women face numerous pressures from careers, family obligations, and societal expectations. This may result in considerable variations in how leisure activities affect health and well-being across different life stages (3, 7). This study therefore aims to analyze the impact of leisure activity participation on health status and subjective well-being among Chinese women at various life stages and explore the mediating role of health status.

Based on existing literature and integrating life course theory, leisure sociology, and subjective well-being frameworks, this study proposes the following hypotheses:

*Hypothesis 1*: Leisure activity participation has a significant positive impact on women's health status.

*Hypothesis 2*: Health status has a significant positive impact on women's subjective well-being.

*Hypothesis 3*: Leisure activity participation indirectly enhances women's subjective well-being by improving health status.

*Hypothesis 4*: The relationship between leisure activity participation, health status, and subjective well-being differs significantly across different life stages of women.

This research will contribute to a deeper understanding of women’s quality of life in Chinese contexts and provide both theoretical support and practical guidance for improving their well-being and health.

## 2 Research methods

### 2.1 Data sources

The data used in this study are derived from the China General Social Survey (CGSS). Initiated in 2003, CGSS is a nationwide, comprehensive, and continuous academic survey project that employs a rigorous stratified random sampling design (13). This survey regularly collects data on various aspects of individual social behavior, health status, educational attainment, social attitudes, and more, while ensuring high scientific rigor and representativeness in the data collection process (14). This study utilizes the 2015 CGSS data, focusing on the female population aged 20 to 55 years. During the data preprocessing phase, cases with missing core variables were excluded, resulting in a final sample of 2,956 valid cases, with an average age of 39.43 ± 10.20 years. This study adopts Gordon’s life stage classification method (15), focusing on three stages of women’s life stages: early adulthood (20–29 years), early mature age (30–44 years), and late mature age (45–retirement). Given that the typical retirement age for women in China is around 55, the study sample consists of adult women aged 20 to 55.

### 2.2 Observed variables

#### 2.2.1 Leisure activity participation

In the CGSS survey, the leisure activity participation section consists of 12 items. To analyze the multidimensional nature of leisure activities, this study employs factor analysis to extract four common factors, which are named as four dimensions of leisure activities:

Physically Active Leisure Activities: Includes items with clear physical activity characteristics, such as shopping and participating in sports exercises (16).

Cultural Leisure Activities: Refers to activities aimed at enhancing personal interests or temperament and improving mental abilities, such as attending cultural events, listening to concerts, viewing exhibitions, or watching competitions (17).

Social Leisure Activities: Focuses on activities that promote interpersonal relationships, such as visiting relatives, making friends, or attending parties.

Recreational Leisure Activities: Primarily involves pure relaxation and entertainment activities in daily life, such as watching TV, browsing the internet, or listening to the radio.

Each dimension is classified according to the nature and goals of the activities, with the number of items in each dimension as follows: two items for physically active leisure activities, three for cultural leisure activities, two for social leisure activities, and five for recreational leisure activities. All items are scored using a five-point Likert scale. The Cronbach’s *α* coefficient of this questionnaire is 0.742, the Kaiser–Meyer–Olkin (KMO) value is 0.835, and Bartlett’s test of sphericity yielded a result of 8012.586 (*p* < 0.01), indicating that the data are suitable for factor analysis. The scores for each dimension are summed to obtain the total score for that dimension, and the sum of all dimension scores represents the overall leisure activity score. Higher scores indicate greater participation in leisure activities.

#### 2.2.2 Health status

Health status is measured by a single question: “How would you rate your current physical health?” Respondents select from the following options based on their own feelings: Very unhealthy = 1; Somewhat unhealthy = 2; Average = 3; Somewhat healthy = 4; Very healthy = 5. For the purpose of analysis, health status is treated as a continuous variable ranging from 1 to 5 for statistical analysis. Studies have shown that it is common to treat health status as a continuous variable in regression analyses (18), which is the approach adopted in this study. Whether using OLS regression analysis or ordered Probit or Logit models, the direction and significance of parameter estimates are consistent (19).

#### 2.2.3 Subjective well-being

Subjective well-being is measured by the question: “Overall, do you feel your life is happy?” Respondents select from the following options based on their actual feelings: Very unhappy = 1; Somewhat unhappy = 2; Neither happy nor unhappy = 3; Somewhat happy = 4; Very happy = 5. Similar to health status, subjective well-being is treated as a continuous variable ranging from 1 to 5 in this study. This approach has been used in multiple well-being studies (20).

#### 2.2.4 Demographic variables

The main demographic variables collected in this study include: age, education level, marital status, household registration, ethnicity, and employment status. Demographic variables are often used as control variables, as studies have shown that these variables can have a potential impact on the relationship between leisure activity participation, health status, and subjective well-being (21). Therefore, these variables are included as control variables in the analysis.

### 2.3 Statistical methods

This study uses SPSS 23.0 software to conduct descriptive analysis, independent samples t-tests, one-way ANOVA, and stepwise regression analysis. For all hypothesis tests, the significance level is set at *p* < 0.05 (22).

## 3 Results

### 3.1 Descriptive characteristics of sample variables

The descriptive analysis results of the observed variables are shown in Table 1.

**Table 1:** Descriptive characteristics of variables.

Variable	Description	Mean	SD
Life cycle stage	20–29 years = 1; 30–44 years = 2; 45–55 years = 3, range 1–3	2.16	0.76
Ethnicity	Han = 1; Ethnic minority = 0, values 0 or 1	0.92	0.27
Education level	Junior high school and below = 1; High school/technical school = 2; Bachelor degree = 3; Graduate degree and above = 4, range 1–4	1.88	0.85
Employment status	Formal employment = 1; No formal employment = 0, values 0 or 1	0.81	0.39
Household registration	Rural household = 1; Non-rural household = 0, values 0 or 1	0.57	0.5
Marital status	Married = 1; Single/divorced = 0, values 0 or 1	0.78	0.42
Health status	Range 1–5, higher value indicates better self-reported health status	3.93	0.97
Subjective well-being	Range 1–5, higher value indicates higher subjective well-being	3.81	0.8
Physical leisure activities	Score, range 2–10	4.89	1.89
Cultural leisure activities	Score, range 3–15	4.53	1.87
Social leisure activities	Score, range 2–10	4.91	1.46
Recreational leisure activities	Score, range 5–25	13.94	3.64
Leisure activity participation	Score, range 12–60	28.26	6.85

### 3.2 Comparison of differences in leisure activity participation, health status, and subjective well-being among women in different life stages

The study found significant differences in Leisure Activity Participation and Health Status among women in different life stages (Table 2). Women in early adulthood exhibited significantly higher levels of participation in various leisure activities compared to those in later stages. The following trends were observed: Physical Leisure Activities (*η*^2^ = 0.078), Cultural Leisure Activities (*η*^2^ = 0.015), Social Leisure Activities (*η*^2^ = 0.135), and Recreational Leisure Activities (*η*^2^ = 0.012) all showed an increasing trend as women transitioned from early adulthood to late maturity. In contrast, overall Leisure Activity Participation (*η*^2^ = 0.006), Health Status (*η*^2^ = 0.023), and Subjective Well-being (*η*^2^ = 0.086) significantly declined with age. The findings highlight the impact of life stages on women’s quality of life, particularly the most notable changes in Health Status and Subjective Well-being.

**Table 2:** Female leisure activity participation, health status, and subjective well-being across different life stages.

Variable	Early adulthood (*n* = 662)	Early maturity (*n* = 1,151)	Late Maturity (*n* = 1,142)	*η* ^2^	*p*-value
Physical leisure activities	6.43 (1.59)	6.68 (1.87)	6.84 (2.27)	0.078	<0.001
Cultural leisure activities	12.75 (1.97)	13.37 (2.10)	13.75 (2.80)	0.015	<0.001
Social leisure activities	6.90 (1.40)	7.18 (1.61)	7.39 (1.88)	0.135	<0.001
Recreational leisure activities	13.49 (3.11)	15.43 (3.87)	17.52 (4.17)	0.012	<0.001
Leisure activity participation	39.58 (5.89)	42.66 (6.97)	45.51 (8.11)	0.006	<0.001
Health status	4.20 (0.78)	3.93 (0.93)	3.49 (1.06)	0.023	<0.001
Subjective well-being	4.14 (0.65)	4.03 (0.76)	3.88 (0.91)	0.086	<0.001

Data are presented as *M*(SD).

### 3.3 Differences in leisure activity participation, health status, and subjective well-being among women in different life stages

The results (Table 3) show that women in the early adulthood stage exhibit significant differences in health status, subjective well-being, and leisure activity participation. Han Chinese women scored slightly higher than women from ethnic minorities in health status (*M* = 4.21 vs. *M* = 4.02) and subjective well-being (*M* = 4.14 vs. *M* = 4.20), but these differences were not statistically significant (health status: *p* = 0.072, subjective well-being: *p* = 0.546). However, Han Chinese women had lower scores in leisure activity participation (*M* = 39.42 vs. *M* = 41.42) compared to women from ethnic minorities, and this difference was statistically significant (*p* = 0.014).

**Table 3:** Comparative analysis of leisure activities, health conditions, and subjective well-being among women in early adulthood stages.

Variable	Health status		Subjective well-being		Total leisure activity score	
	*M*(SD)	*p*-value	*M*(SD)	*p*-value	*M*(SD)	*p*-value
Ethnicity		0.072		0.546		0.014
Minorities	4.02 (0.88)		4.20 (0.63)		41.42 (6.85)	
Han	4.21 (0.77)		4.14 (0.65)		39.42 (5.78)	
Education level		0.065		0.015		<0.001
Junior High and Below	4.11 (0.86)		4.06 (0.64)		42.97 (6.14)	
High School/Technical School	4.13 (0.82)		4.06 (0.78)		40.07 (4.25)	
Bachelor Degree	4.28 (0.72)		4.21 (0.60)		37.16 (5.07)	
Graduate Degree and Above	4.29 (0.60)		4.35 (0.49)		35.93 (3.61)	
Household Registration		0.617		0.247		<0.001
Rural	4.18 (0.81)		4.12 (0.65)		40.86 (5.87)	
Non-rural	4.22 (0.74)		4.18 (0.64)		37.67 (5.39)	
Employment Status		0.264		0.894		0.030
Unemployed	4.16 (0.84)		4.14 (0.65)		40.62 (6.13)	
Employed	4.23 (0.73)		4.15 (0.65)		38.63 (5.51)	
Marital Status		0.087		0.895		< 0.001
Single/Divorced	4.25 (0.79)		4.14 (0.70)		37.58 (5.22)	
Married	4.15 (0.78)		4.15 (0.61)		41.11 (5.93)	

Data are presented as *M*(SD).

Women with higher educational attainment showed significant improvements in health status, subjective well-being, and leisure activity participation. Specifically, women with a graduate degree or higher reported the best health status (*M* = 4.29) and subjective well-being (*M* = 4.35). Education level had a significant impact on leisure activity participation (*p* < 0.001), with women with junior high school education or below showing the highest level of participation (*M* = 42.97). As education level increased, leisure activity participation gradually decreased, with women holding graduate degrees or higher having the lowest participation (*M* = 35.93). However, education level had only a marginal effect on health status (*p* = 0.065).

Urban women outperformed rural women in health status (*M* = 4.22 vs. *M* = 4.18, *p* = 0.028) and leisure activity participation (*M* = 37.67 vs. *M* = 40.86, *p* < 0.001). However, no significant difference was found between urban and rural women in terms of subjective well-being (*p* = 0.247).

Employed women showed slightly better health status (*M* = 4.23 vs. *M* = 4.16) than those who were not employed, although there was no significant difference in subjective well-being (*M* = 4.15 vs. *M* = 4.14) between the two groups (*p* = 0.264 and *p* = 0.894). However, women who were not employed had a slightly higher level of leisure activity participation (*M* = 40.62 vs. *M* = 38.63), and employment status significantly affected leisure activity participation (*p* = 0.030).

Married women had significantly higher leisure activity participation than unmarried women (*M* = 41.11 vs. *M* = 37.58, *p* < 0.001), but there was no significant difference between the two groups in terms of health status (*p* = 0.087) and subjective well-being (*p* = 0.895). Marital status had a significant impact on leisure activity participation (*p* < 0.001).

Overall, education level, household registration type, employment status, and marital status significantly influenced women’s leisure activity participation, and significant differences in health status were also found between urban and rural women.

The results (Table 4) show that women in the early middle-aged stage exhibit differences in health status, subjective well-being, and leisure activity participation. The key findings are as follows:

**Table 4:** Comparative analysis of leisure activities, health conditions, and subjective well-being among women in early maturity period.

Variable	Health status		Subjective well-being		Total leisure activity score	
	*M*(SD)	*p*-value	*M*(SD)	*p*-value	*M*(SD)	*p*-value
Ethnicity		0.002		0.116		0.010
Minorities	3.68 (0.98)		3.91 (0.78)		44.21 (6.90)	
Han	3.96 (0.93)		4.04 (0.76)		42.51 (6.96)	
Education level		<0.001		0.013		<0.001
Junior High and Below	3.82 (1.00)		3.97 (0.83)		45.37 (6.49)	
High School/Technical School	4.11 (0.84)		4.05 (0.73)		39.84 (5.82)	
Bachelor Degree	4.10 (0.79)		4.15 (0.55)		37.72 (5.26)	
Graduate Degree and Above	3.87 (0.81)		4.00 (0.76)		39.13 (5.84)	
Household Registration		0.014		0.160		<0.001
Rural	3.88 (0.97)		4.00 (0.78)		44.84 (6.91)	
Non-rural	4.00 (0.88)		4.06 (0.73)		39.90 (6.00)	
Employment Status		<0.001		0.040		<0.001
Unemployed	4.07 (0.84)		3.86 (0.76)		28.68 (6.43)	
Employed	3.71 (1.13)		3.70 (0.90)		26.12 (6.41)	
Marital Status		0.172		<0.001		0.061
Single/Divorced	3.80 (1.08)		3.58 (1.07)		41.35 (6.31)	
Married	3.94 (0.92)		4.05 (0.73)		42.76 (7.01)	

Data are presented as *M*(SD).

Statistically significant differences were observed between Han Chinese women and women from ethnic minorities in health status (*p* = 0.002), subjective well-being (*p* = 0.116), and total leisure activity participation (*p* = 0.010). Specifically, women from ethnic minorities scored higher in health status (*M* = 3.68) and total leisure activity participation (*M* = 44.21), but the difference in subjective well-being was not significant (*p* = 0.116). Thus, ethnic factors had a larger impact on health status and leisure activity participation, but a relatively smaller effect on subjective well-being.

Women with higher educational attainment performed significantly better than other educational groups in health status, subjective well-being, and total leisure activity participation, especially those with a graduate degree or higher. Specifically, women with junior high school education or below performed the best in health status (*M* = 3.82), subjective well-being (*M* = 3.97), and leisure activity participation (*M* = 45.37) (*p* < 0.001). However, as education level increased, health status and subjective well-being scores gradually stabilized, while leisure activity participation slightly declined.

Urban women scored significantly higher than rural women in subjective well-being (*p* < 0.01) and total leisure activity participation (*p* < 0.001), with *M* = 4.06 and *M* = 39.90, respectively, compared to *M* = 4.00 and *M* = 44.84 for rural women. Although household registration type had an effect on health status (*p* = 0.014), the difference was relatively small.

Employed women performed significantly better than unemployed women in health status (*M* = 3.71 vs. *M* = 4.07, *p* < 0.001) and subjective well-being (*M* = 3.70 vs. *M* = 3.86, *p* = 0.040). However, unemployed women had slightly higher leisure activity participation (*M* = 28.68 vs. *M* = 26.12), and employment status had a significant impact on leisure activity participation (*p* < 0.001).

Married women had significantly better subjective well-being (*M* = 4.05 vs. *M* = 3.58, *p* < 0.001) than unmarried or divorced women, but no significant differences were found between the two groups in health status (*p* = 0.172) and leisure activity participation (*p* = 0.061).

Overall, education level, household registration type, employment status, and marital status had a significant impact on women’s health status, subjective well-being, and leisure activity participation, while ethnic factors had a relatively smaller influence.

The results (Table 5) reveal significant differences in health status, subjective well-being, and leisure activity participation among women in the late middle-aged stage. The key findings are as follows:

**Table 5:** Comparative analysis of leisure activities, health conditions, and subjective well-being among women in late maturity period.

Variable	Health status		Subjective well-being		Total leisure activity score	
	*M*(SD)	*p*-value	*M*(SD)	*p*-value	*M*(SD)	*p*-value
Ethnicity		<0.001		0.030		0.001
Minorities	3.12 (1.19)		3.71 (1.04)		47.75 (7.81)	
Han	3.53 (1.03)		3.90 (0.89)		45.29 (8.10)	
Education Level		<0.001		0.001		<0.001
Junior High and Below	3.43 (1.10)		3.83 (0.94)		47.08 (7.95)	
High School/Technical School	3.68 (0.91)		4.03 (0.76)		41.64 (6.21)	
Bachelor Degree	3.76 (0.79)		4.15 (0.72)		37.14 (5.03)	
Graduate Degree and Above	3.60 (0.55)		4.40 (0.55)		38.60 (7.02)	
Household Registration		0.144		0.812		<0.001
Rural	3.59 (1.07)		3.73 (0.83)		23.39 (5.50)	
Non-rural	3.68 (1.00)		3.74 (0.82)		29.23 (6.09)	
Employment Status		<0.001		0.004		<0.001
Unemployed	3.37 (1.09)		3.83 (0.95)		46.80 (8.24)	
Employed	3.73 (0.94)		3.99 (0.79)		43.03 (7.23)	
Marital Status		0.006		<0.001		0.206
Single/Divorced	3.26 (1.05)		3.37 (1.24)		44.70 (8.41)	
Married	3.52 (1.05)		3.94 (0.84)		45.60 (8.07)	

Data are presented as M(SD).

There are significant differences between Han Chinese women and women from ethnic minorities in terms of health status (*p* < 0.001), subjective well-being (*p* = 0.030), and overall leisure activity participation (*p* = 0.001). Specifically, women from ethnic minorities scored lower in health status (*M* = 3.12) and subjective well-being (*M* = 3.71), but higher in leisure activity participation (*M* = 47.75) compared to Han Chinese women (*M* = 45.29).

Women with higher educational attainment demonstrated significantly better outcomes in health status, subjective well-being, and leisure activity participation, especially those with graduate degrees or higher. Specifically, women with junior high school education or below had the highest scores in health status (*M* = 3.43), subjective well-being (*M* = 3.83), and leisure activity participation (*M* = 47.08) (*p* < 0.001). However, as education levels increased, health status and subjective well-being scores became more stable, while leisure activity participation slightly decreased among women with graduate degrees or higher (*M* = 38.60).

Urban women showed significantly higher scores than rural women in subjective well-being (*p* < 0.01) and overall leisure activity participation (*p* < 0.001), with urban women scoring *M* = 4.06 and *M* = 39.90, respectively, compared to *M* = 4.00 and *M* = 44.84 for rural women. Although household registration type did influence health status (*p* = 0.014), the difference was relatively small.

Employed women had significantly higher scores in health status (*M* = 3.73 vs. *M* = 3.37, *p* < 0.001) and subjective well-being (*M* = 3.99 vs. *M* = 3.83, *p* = 0.004) than unemployed women. However, unemployed women had slightly higher leisure activity participation (*M* = 46.80 vs. *M* = 43.03), and employment status had a significant impact on leisure activity participation (*p* < 0.001).

Married women exhibited significantly better health status (*M* = 3.52 vs. *M* = 3.26, *p* = 0.006) and subjective well-being (*M* = 3.94 vs. *M* = 3.37, *p* < 0.001) compared to unmarried or divorced women. However, no significant difference was observed between these two groups in terms of leisure activity participation (*p* = 0.206).

In conclusion, education level, employment status, and marital status are significant factors influencing health status, subjective well-being, and leisure activity participation in the late middle-aged stage, while ethnic and household registration types have relatively less influence on these factors.

### 3.4 The impact of leisure activity participation and health status on women’s subjective well-being across different life stages

Through stepwise regression analysis, the impact of leisure activity participation and health status on women’s subjective well-being at different stages of the life cycle was explored. The results indicate significant differences in the influence of these factors on subjective well-being across various stages.

In early adulthood (Table 6), marital status (*β* = 0.209, *p* < 0.01), recreational leisure activities (*β* = 0.16, *p* < 0.01), and health status (*β* = 0.165, *p* < 0.01) all significantly and positively influenced subjective well-being.

**Table 6:** Effects of leisure activities and health conditions on subjective well-being among women in early adult stage.

Variable	Coefficient	SE	*t*	*p*-value
Recreational leisure activity	0.032	0.012	2.48	0.013
Health status	0.097	0.030	3.19	0.001

In early mature age (Table 7), marital status (*β* = 0.214, *p* = 0.028), social leisure activities (*β* = 0.047, *p* = 0.004), health status (*β* = 0.096, *p* < 0.001), and employment status (*β* = 0.282, *p* < 0.001) all significantly and positively influenced subjective well-being.

**Table 7:** Effects of leisure activities and health conditions on subjective well-being among women in early mature period.

Variable	Coefficient	SE	*t*	*p*-value
Marital status	0.214	0.097	2.20	0.028
Employment status	0.282	0.055	5.05	<0.001
Ethnicity	0.238	0.085	2.80	0.005
Social leisure activities	0.047	0.016	2.91	0.004
Health status	0.096	0.017	5.39	<0.001

In late mature age (Table 8), marital status (*β* = 0.294, *p* = 0.001), physical leisure activities (*β* = 0.045, *p* = 0.001), health status (*β* = 0.254, *p* < 0.01), and employment status (*β* = 0.263, *p* < 0.001) had a significant positive effect on subjective well-being, and ethnic factors (*β* = 0.331, *p* = 0.001) also had a significant impact.

**Table 8:** Effects of leisure activities and health conditions on subjective well-being among women in late mature period.

Variable	Coefficient	SE	*t*	*p*-value
Marital status	0.294	0.090	3.24	0.001
Employment status	0.263	0.063	4.15	<0.001
Ethnicity	0.331	0.095	3.46	0.001
Physical leisure activities	0.045	0.014	3.25	0.001
Health status	0.080	0.023	3.47	0.001

### 3.5 The role of health status in the mechanism through which leisure activity participation affects subjective well-being in adult women

Health status significantly predicts the subjective well-being of adult women across different life stages. Research has shown that as leisure activity participation increases, the subjective well-being of adult women improves positively. Thus, the question arises: does leisure activity participation indirectly influence subjective well-being through the improvement of health status?

The findings of this study show that leisure activities have a significant impact on health status. The regression analysis results indicate that the path coefficient for leisure activities influencing health status is 0.196 (*z* = 10.869, *p* < 0.01), revealing a significant positive relationship between leisure activities and health status. Moreover, health status significantly contributes to subjective well-being, with a path coefficient of 0.234 (*z* = 13.050, *p* < 0.01), indicating that health status positively influences subjective well-being.

Regarding the direct effects, the path coefficient for leisure activities on subjective well-being is 0.146 (*z* = 8.155, *p* < 0.01), further confirming the positive effect of leisure activities on subjective well-being (see Table 9).

**Table 9:** Model regression coefficients.

Path	Non-standardized coefficient	*Z*	SE	*p*-value	Standardized coefficient
Leisure Activity → Health Status	0.028	10.869	0.003	<0.001	0.196
Health Status → Subjective Well-being	0.192	13.05	0.015	<0.001	0.234
Leisure Activity → Subjective Well-being	0.017	8.155	0.002	<0.001	0.146

Further analysis of the mediating role of health status revealed that health status partially mediates the relationship between leisure activity participation and subjective well-being. Specifically, leisure activities first significantly improve health status and then indirectly enhance subjective well-being through the improvement of health status. This result suggests that increasing leisure activity participation not only directly promotes the physical and mental health of adult women but also significantly improves their subjective well-being through health status improvements.

## 4 Discussion

### 4.1 Dynamic changes in leisure activity participation from a lifespan perspective

This study reveals significant differences in women’s Leisure Activity Participation across various life stages, with a systematic decline observed in all types of leisure engagement as age increases. Women in early adulthood demonstrate the highest overall participation level (32.42 ± 6.09), which declines substantially in late adulthood (25.77 ± 6.42). These trends can be interpreted from dual perspectives: developmental psychology, which emphasizes the changing cognitive and emotional capacities across the lifespan, and social role theory, which considers the evolving societal expectations and responsibilities assigned to women.

Different categories of leisure activities exhibit distinct patterns of change. Participation in Cultural Leisure Activities shows the most pronounced decline (from 5.52 ± 2.17 to 3.93 ± 1.49), likely reflecting a reallocation of time resources prompted by shifting social roles. Engagement in Physical Leisure Activities also significantly decreases (from 5.58 ± 1.66 to 4.62 ± 1.98), aligning with age-related physiological changes and the principle of energy prioritization. In contrast, Social Leisure Activities experience a relatively smaller reduction (from 5.36 ± 1.41 to 4.62 ± 1.45), underscoring the enduring nature of social connectedness as a basic psychological need. Similarly, participation in Recreational Leisure Activities declines markedly (from 15.97 ± 2.95 to 12.60 ± 3.59), further substantiating the age-related decline in overall Leisure Activity Participation.

These findings are consistent with life course theory, which posits that individuals dynamically adjust their allocation of time and energy across life stages (23). During early adulthood, women often face fewer role constraints and have greater discretionary time, enabling more active and diversified leisure participation. Research indicates that occupational autonomy and social network expansion are key drivers of such engagement during this stage (24).

As women transition into midlife, the cumulative burden of family responsibilities, childrearing, and career progression significantly reduces their available time for leisure. In late adulthood, compounded challenges such as caregiving obligations and Health Status concerns further constrain leisure engagement. These patterns align with existing findings on Leisure Activity Participation in middle-aged and older populations (25). Additionally, increased responsibility for elder care further diminishes personal leisure time, reinforcing the effects of role transitions and social expectations in shaping women’s leisure trajectories over the life course (26).

### 4.2 Lifespan changes in women’s health status

The results of this study indicate a marked decline in women’s Health Status across the life course, decreasing from early adulthood (4.28 ± 0.82) to late adulthood (3.63 ± 1.04), a trend that closely parallels the observed reduction in Leisure Activity Participation. This deterioration in health can be attributed to a confluence of biological and psychosocial factors. On the one hand, age-related physiological decline and the increased incidence of chronic diseases serve as fundamental contributors. On the other hand, the long-term physical impact of reproductive history, coupled with the cumulative stress associated with multiple social role transitions, exerts additional strain (27).

Consistent with these findings, prior literature has firmly established the link between age-related biological changes and declining Health Status. Of particular concern in late adulthood are female-specific health challenges, including menopausal symptoms, hormonal fluctuations, and reductions in bone mineral density. These changes have far-reaching consequences, affecting not only physical functioning but also mental health outcomes (28). Epidemiological studies further corroborate this pattern, showing a sharp rise in the prevalence of chronic conditions such as hypertension, diabetes, and arthritis among aging women, which forms a critical clinical basis for deteriorating health (29).

From a biosocial perspective, the age-related gradient in women’s Health Status reflects the intricate interplay between biological and social determinants. Postmenopausal hormonal shifts may directly impair physiological regulation and indirectly affect psychological well-being by altering emotional regulation capacity (30). Concurrently, the increased burden of social roles alongside dwindling personal resources generates psychosocial stress, potentially impacting overall health through stress-response pathways (31). The parallel trajectories of declining health and reduced Leisure Activity Participation suggest that leisure behaviors may serve as a critical mediating mechanism linking life stage to health outcomes.

### 4.3 Changes in subjective well-being across the lifespan

The data from this study demonstrate a modest age-related decline in women’s Subjective Well-being, decreasing from 3.90 ± 0.77 in early adulthood to 3.73 ± 0.83 in late adulthood. Although this change is statistically significant (*F* = 10.67), its magnitude is notably smaller than the sharp decline observed in Health Status (*F* = 110.45). This contrast highlights the complex interplay among Subjective Well-being, Leisure Activity Participation, and Health Status, and suggests that women may possess robust psychological adaptation mechanisms across the life span.

From a developmental psychology perspective, the relative stability of Subjective Well-being may stem from the synergistic effects of multiple psychological and environmental factors (32). In early adulthood, women benefit from professional advancement and expanding social networks, which foster high levels of well-being (33). As they progress into midlife and beyond, despite facing dual pressures from work–family balance and gradual physiological changes, these potentially adverse factors are often offset by enhanced psychological coping strategies and accumulated life wisdom (34–36).

These findings align closely with the set-point theory of well-being, which posits that despite substantial environmental changes, individuals’ Subjective Well-being tends to fluctuate around a personal baseline and eventually returns to it (37). This theory underscores the central role of psychological resilience in maintaining stable well-being, particularly during life stage transitions (38). Research has shown that women often exhibit strong cognitive reappraisal abilities, enabling them to reconstruct the meaning of life events and thereby preserve emotional balance and life satisfaction.

From a social psychological viewpoint, several psychosocial resources—such as the quality of social support networks, accumulated experiential wisdom, and enhanced self-efficacy—act as critical protective factors for women’s Subjective Well-being in mid-to-late adulthood (39). The effective mobilization of these resources may explain why women can maintain a relatively stable level of well-being despite significant declines in Health Status. Another key explanatory factor lies in the dynamic reconfiguration of values and life expectations (40). As women age, they increasingly shift the sources of their well-being from external achievements and physical vitality to emotional connectedness and a sense of life meaning (41). This internal value orientation serves as a psychological buffer against external stressors.

At a practical level, effective social integration strategies encompass a range of supportive structures: intergenerational family activities that reinforce belonging through reciprocal relationships with children and grandchildren; maintenance of diverse friendship networks, particularly cross-generational ties; participation in community volunteer services that enhance self-worth through prosocial behavior; and group affiliations based on shared interests or beliefs, which provide value affirmation and spiritual support. Collectively, these mechanisms offer emotional, informational, and instrumental support, constituting a foundational system for sustaining and enhancing women’s Subjective Well-being.

In sum, these findings underscore the importance of a holistic approach to promoting women’s well-being throughout the life course. Equal emphasis should be placed on physical health maintenance, Leisure Activity Participation, and the optimization of psychosocial resources. Tailored social support and resilience-building interventions, particularly during transitional periods, are essential for fostering coordinated physical and psychological health.

### 4.4 Complex mechanisms between leisure activity, health status, and subjective well-being

The path analysis conducted in this study reveals a complex and systematic network of associations among Leisure Activity Participation, Health Status, and Subjective Well-being. The data demonstrate that Health Status plays a critical partial mediating role in the relationship between leisure engagement and well-being. Specifically, Leisure Activity Participation not only exerts a direct positive effect on Subjective Well-being (*β* = 0.31, *p* < 0.01) but also indirectly enhances well-being by improving Health Status (*β* = 0.37, *p* < 0.001; indirect effect *β* = 0.25, *p* < 0.01). This multipath mechanism aligns closely with previous findings, underscoring the multifaceted contributions of leisure activities to women’s physical and psychological health (42).

From a physiological perspective, different types of leisure activities influence Health Status through distinct mechanisms. Physical Leisure Activities—such as aerobic exercise performed 3–4 times per week for at least 30 min—significantly improve cardiorespiratory function, enhance muscle strength, increase bone density, and optimize metabolic parameters. These changes are directly linked to reduced health risks common among middle-aged and older women, such as cardiovascular disease and osteoporosis (43, 44). Research suggests that regular participation in moderate-intensity physical activity can lower cardiovascular disease risk by approximately 30–40% in midlife women (45).

Social Leisure Activities, such as participating in community group events 1–2 times per week, enhance health by alleviating loneliness, offering emotional support, and reducing psychological stress (46). Physiological studies have confirmed that active social engagement reduces levels of inflammatory biomarkers (e.g., IL-6 and C-reactive protein), promotes immune competence, and improves endocrine regulation (47). Longitudinal data from mature adult women further indicate that those with high-quality social networks exhibit smaller cortisol fluctuations and superior immune profiles—factors strongly associated with better Health Status and delayed aging (48).

Cultural Leisure Activities, including weekly participation in reading, creative writing, or the arts, primarily benefit Health Status through cognitive stimulation and emotional regulation (49). Neuroscientific research indicates that continuous cognitive engagement fosters neural plasticity and strengthens cognitive reserve, thereby slowing age-related cognitive decline (50). Moreover, the positive affective experiences commonly elicited by cultural activities modulate neuroendocrine function, reduce stress hormone levels, and enhance autonomic nervous system balance—all of which are associated with improved immune function and inflammatory control (51).

Importantly, both the frequency of activity participation and the surrounding social context play pivotal roles in health promotion. Studies show that regular involvement in community-based leisure is associated with improved mental health, greater social integration, and sustained functional capacity among older adults (46). Furthermore, group-based activities appear to produce more substantial health benefits than solitary leisure pursuits, likely due to the added psychophysiological advantages of social interaction.

Improvements in Health Status in turn exert a positive influence on Subjective Well-being, establishing a reinforcing cycle. Enhanced physical health reduces discomfort and functional limitations, thereby expanding women’s activity range and autonomy. Improvements in psychological health directly elevate emotional quality and life satisfaction. Empirical evidence suggests that women who experience health gains as a result of leisure engagement report a 15–20% increase in Subjective Well-being on average (56).

In summary, these findings highlight the integral role of leisure engagement as a multidimensional mechanism for health promotion and well-being enhancement, particularly during transitional phases in the female life course. The synergistic participation in diverse forms of Leisure Activities may yield optimal health and happiness outcomes, offering a robust theoretical foundation for designing targeted health-promotion interventions.

### 4.5 Moderating role of demographic characteristics

This study found that demographic characteristics such as education, employment status, and marital status significantly influence women’s participation in leisure activities, health status, and subjective well-being. These results are consistent with numerous studies that emphasize the critical role of education, occupation, and social support in individual quality of life (52). Women with higher levels of education, those employed, and those married tend to perform better across these dimensions, underscoring the importance of social resources and support for quality of life.

For example, prior research has shown that women with higher education typically have more economic resources and social support, which enables them to achieve better quality of life and health outcomes (53). Similarly, married women often have more family support, which positively impacts their physical and mental health (54). Furthermore, employment status plays an important role in quality of life. Employed women often enjoy economic independence and social recognition, leading to higher levels of subjective well-being (55).

### 4.6 Study limitations and future directions

While this study offers important insights into the interrelationships among Leisure Activity Participation, Health Status, and Subjective Well-being across the female life course, several notable limitations warrant consideration. First, the cross-sectional design precludes definitive conclusions regarding the causal directionality of the observed associations, thereby constraining the generalizability of the findings. Second, the sample exhibited homogeneity in terms of geographic distribution, educational attainment, and socioeconomic background, and the unequal sample sizes across age groups may have affected the sensitivity of the statistical analyses. Third, the study did not sufficiently account for cultural context, thus limiting the exploration of how cultural variables may shape leisure patterns and the construction of well-being. In addition, reliance on self-report measures may introduce bias due to social desirability effects. The measurement of Leisure Activity Participation focused primarily on frequency rather than quality or subjective meaningfulness. Furthermore, other potentially important mediating or moderating variables were not included in the analytical framework.

Future research should adopt longitudinal designs to establish temporal and causal relationships among Leisure Activity Participation, Health Status, and Subjective Well-being. Stratified random sampling strategies should be employed to ensure more balanced representation of women from diverse demographic and socioeconomic backgrounds. Cross-cultural comparative studies are needed to examine the moderating influence of cultural norms and values. Integrating both objective and subjective indicators could improve measurement precision. Finally, more comprehensive theoretical models should be developed to incorporate additional psychosocial variables as potential mediators or moderators. By addressing these limitations, future studies can not only deepen our understanding of women’s developmental trajectories but also provide more robust scientific evidence to inform health promotion strategies tailored to the needs of women across the life course.

## 5 Conclusion

This study explored the dynamic changes in women’s participation in leisure activities, health status, and subjective well-being across different life stages from a lifespan perspective. The findings indicate that as women age, their health status and subjective well-being significantly decline, particularly in late middle age. However, women in late middle age showed the highest participation in leisure activities. Leisure activities indirectly enhance subjective well-being by improving health, and demographic characteristics such as education, employment status, and marital status play important moderating roles in women’s quality of life. These findings provide theoretical support for policymakers, emphasizing the importance of providing appropriate leisure activities and health promotion interventions for women at different stages of life. Future research could further explore the long-term dynamic changes of these factors and their underlying mechanisms.

## Data Availability

The datasets presented in this study can be found in online repositories. The names of the repository/repositories and accession number(s) can be found: http://www.cgss.cn/.
